# Early detection of myocardial involvement in idiopathic inflammatory myopathies detected by cardiac magnetic resonance imaging

**DOI:** 10.1186/1532-429X-17-S1-P277

**Published:** 2015-02-03

**Authors:** Dominik Buckert, Angela Rosenbohm, Jan Kassubek, Albert Ludolph, Peter Bernhardt

**Affiliations:** Innere Medizin II, Uniklinik Ulm, Ulm, Germany; Neurology, Uniklinik Ulm, Ulm, Germany

## Background

Idiopathic inflammatory myopathies are heterogeneous systemic autoimmune diseases that are characterized by progressive muscle weakness and typical inflammatory infiltration in skeletal muscle. It is known that a cardiac involvement may occur; the frequency varies between 9% and 72%, mainly depending on patient selection and diagnostic procedures applied. Most common finding is a progressive (systolic) heart failure. Diagnosis may be difficult in patients that exhibit exercise limitation due to peripheral muscle weakness.

It has been shown that cardiac magnetic resonance imaging (CMR) is reliably able to provide important information concerning cardiac function and inflammatory processes in a single exam. Therefore, objective of our prospective study was to assess and characterize myocardial involvement in patients with histological defined idiopathic inflammatory myopathies by a comprehensive CMR protocol.

## Methods

Fifty-three consecutive patients with histologically proven idiopathic inflammatory myopathy were enrolled into the study.

All patients underwent CMR examination in a 1.5-T whole body scanner using a 32-channel phased-array cardiac surface coil. Volumetric and functional assessment was performed using 3D short-axis steady-state free precession sequences covering the entire left and right ventricle. Early myocardial enhancement was assessed before and shortly after the admission of gadolinium based contrast agent by T1-weighted fast spin-echo sequences. Inversion-recovery gradient-echo sequences were acquired for the evaluation of late gadolinium enhancement (LGE) 10-15 minutes after administration of 0.2 mmol/kg gadolinium based contrast agent (Dotarem, Guerbet, France).

CMR images were analyzed by two experienced and blinded readers in consensus.

## Results

Reduced left ventricular function (ejection fraction < 60%) was present in 9 (17%) patients. These patients exhibited significantly higher blood levels of CK-MB (283±193 vs. 175±221 U/l, p=0.02) than patients with preserved LVEF. Early myocardial enhancement (>45% signal intensity increase) was more often present (6 out of 9 [66.7%] vs. 25 out of 44 [56.8%], p=.014) as well as LGE patterns consistent with myocardial inflammation (9 out of 9 [100%] vs. 24 out of 44 [54.5%], p=.001). LGE was most often observed in the inferior and lateral segments.

## Conclusions

The results of this study show that CMR is able to detect cardiac involvement in idiopathic inflammatory myopathies. Early diagnosis using CMR could result in early therapy of heart failure, leading to beneficial ventricular remodeling and thus improving quality of life and survival in these patients.

